# Properties and clinical application safety of antibiotic-loaded bone cement in kyphoplasty

**DOI:** 10.1186/s13018-019-1200-3

**Published:** 2019-07-27

**Authors:** Maciej Opalko, Hans Bösebeck, Sebastian Vogt

**Affiliations:** 1 Klinik für Orthopädie und Unfallchirurgie, Kreiskrankenhaus Prenzlau, Prenzlau, Germany; 2 Heraeus Medical GmbH, Medical Affairs, Philipp-Reis-Str. 8/13, 61273 Wehrheim, Germany; 3 Heraeus Medical GmbH, Innovation, Philipp-Reis-Str. 8/13, 61273 Wehrheim, Germany

**Keywords:** Antibiotic-loaded bone cement (ALBC), Local antibiotic treatment, Kyphoplasty, Gentamicin, Osteopal G, Low viscosity cement, PMMA

## Abstract

**Background:**

Evidence on antibiotic-loaded bone cement remains too vague to guide kyphoplasty in patient care. We clinically evaluated the properties and benefits of a new low viscosity polymethylmethacrylate (PMMA) bone cement loaded with gentamicin.

**Methods:**

In this non-randomised, monocentric, prospective open trial, 50 consecutively enrolled patients with fractures of the vertebral body (TH7-L4) due to osteoporosis or trauma were investigated between 2010 and 2013, with a 1-year post-op follow-up per patient. The antibiotic-loaded PMMA bone cement was administered to patients during the surgery according to the standard procedure established on site for one-staged kyphoplasty. The clinical outcome was assessed according to function and pain by standardised anamnesis, clinical investigation, validated visual analogue scale (VAS) vertebral spine score, Oswestry Low-Back-Pain (ODI) Disability score, and Short Form (SF)-36 score. We further performed X-ray and magnetic resonance imaging with radiomorphometric assessment.

**Results:**

The patients showed beneficial effects concerning low back pain disability (mean ODI score; screening, 68.0 ± 15.8% vs month 12, 42.8 ± 24.5%). The pain level was decreased (VAS vertebral spine score; screening, 68.8 ± 17.6 vs month 12, 43.8 ± 22.2) and the general health state was improved (SF-36; especially ‘role limitations due to emotional problems’ (51.9 ± 44.7; month 6), followed by ‘role limitations due to physical health’ (36.1 ± 42.4; month 6), and ‘pain’ (34.6 ± 35.3; month 6)). No vertebral infection did occur during the hospital stay or the 1-year follow-up. The stabilisation and restoration of the fractured bodies were radiologically confirmed. A reduced rate of leakage was observed, combined with a decreased risk of infection and an improved patient safety after a 1-year follow-up period.

**Conclusion:**

Requirements for bone cement in a kyphoplasty setting were excellently fulfilled. Application technique and cement properties may influence the success of the surgery.

**Trial registration:**

Deutsche Institut für Medizinische Dokumentation und Information (DIMDI), HM-KS-0901, Registered 14 September 2009, https://www.dimdi.de/dynamic/de/medizinprodukte/datenbankrecherche/

## Background

Fractures of the vertebral body have a prevalence of approximately 10–15% in patients over 50 years of age. They may be treated by a vertebroplasty and/or kyphoplasty as accepted procedures in this clinical setting [1–3]. Kyphoplasty provides a minimally invasive method for the stabilisation of fractured vertebral bodies with the largest extent of decompression through a balloon system. The technique has been widely used since the year 2000 [4]. Compared to vertebroplasty [5], kyphoplasty shows benefits regarding the quality of life [6] and reveals an equal or improved efficacy in restoring the mechanical function after severe vertebral wedge fractures.

However, along with an increase in the total number of kyphoplasties, the rate of infections after the application of cement has increased as well [7]. Spondylitis caused by foreign bodies appears to be a severe complication in this group of patients [8]. Recent experience with hip endoprosthesis has shown that adding the antibiotic gentamicin [9] to the bone cement used during the surgery is an appropriate approach to decrease the rate of perioperative infections [10]. Such an effect can also be expected in kyphoplasty when using a gentamicin-loaded cement with proper handling characteristics, including an adequate working phase with constant viscosity. Furthermore, a comparable or better rate of cement leakage [11, 12], pain reduction, and a radiologically confirmed reconstruction of the height of the vertebral body may be achieved in kyphoplasty by using a gentamicin-loaded cement with suitable viscosity.

The use of antibiotic-loaded bone cement (ALBC) for the treatment of fractures of the vertebral body in a kyphoplasty setting is currently not well supported by evidence through literature [13]. Key questions in dealing with ALBCs are a better understanding of the cement properties, application benefits, and safety for clinical use. To overcome this lack of information, prospective trials with ALBCs are needed to investigate both efficacy and risk.

The primary objective of this prospective clinical trial was to observe the properties of a low-viscosity, antibiotic-loaded bone cement in a kyphoplasty setting. Secondary objectives were clinical outcome, application safety, and influence of the administered cement on the success of the surgery [1].

## Materials and methods

### Study design

We clinically evaluated properties and benefits of a new low viscosity polymethylmethacrylate (PMMA) bone cement loaded with gentamicin in a non-randomised, monocentric, prospective open trial conducted in a German centre from 2010 to 2013. In total, 50 patients undergoing a one-staged kyphoplasty were enrolled. As there was no control group, only descriptive statistical methods were used.

### Patient population

The trial population comprised of male or female patients of at least 50 years of age. In total, 50 patients with fractures of the vertebral body (TH7-L4) due to osteoporosis [14] or a trauma scheduled for one-staged kyphoplasty were consecutively screened and enrolled, and 49 were included in the safety population (SAF population). Exclusion criteria were known hypersensibility against ingredients of the tested bone cement, concomitant systemic gentamicin treatment, any concomitant disease (excluding a minimally invasive kyphoplasty of the vertebral body), presence of spondylitis or infection, renal impairment, sensorineural hearing loss, and hemorrhagic diathesis.

All patients who fulfilled the inclusion and exclusion criteria gave their written consent to participate. The trial duration per patient consisted of a screening period up to 7 days before the surgery, a hospitalisation period of 1 week including final examination, and a follow-up period of 12 months. The trial comprised ten clinical visits.

### Study intervention

The investigational medical product (IMD) was administered to patients during the surgery according to the standard procedure established on site for one-staged kyphoplasty [15]. The antibiotic-loaded bone cement consists of two components—a powder and a fluid—and was mixed directly before use (Osteopal G, Heraeus Medical GmbH, Wehrheim, Germany) [16, 17]. The powder contained 0.325 g gentamicin per 26.5 g pouch of bone cement, and the fluid consisted of methyl methacrylate, dimethyl-p-toluidine, hydroquinone, and colorant E 141 in an ampoule of 10 ml. It was applied intra-operatively for minimal invasive kyphoplasty. The right time for the application was governed through the cement polymerization status. Depending on the need for kyphoplasty bone void filling of the vertebral body after fractures, the dosage was selected with usually 3 ml bone cement per level. It was administered as a single intra-operative injection.

Radiomorphometric evaluations of native two-level X-ray images were performed by two independent clinicians using six landmarks to determine the following medical parameters: the angle of kyphosis—the angle between two tangents attached to the ground and endplate of the radiological sagittal parameters of the spine—and the vertebral body index—the quotient of height of leading and rear edge and the pre- and post-operative height of the centre of the vertebral body (in %). As a reference (100%), the median height of an adjacent non-fractured vertebral body was used.

### Study measures

The primary endpoints were the evaluation of the function by pain scores such as the visual analogue scale (VAS) vertebral spine score [18], the Oswestry-Low-Back-Pain-Disability (ODI) score [19], and the Short Form (SF)-36 Health Status Query [20]. They were measured at up to 7 days before the surgery, during the final examination on day 6, and at 6 and 12 months after the surgery. The radiological examination comprised two-level X-ray-radiographs before the surgery, on the day of the surgery, and during the final examination as well as during the follow-ups at 6 and 12 months and magnetic resonance imaging radiomorphometry [21] of the augmented region preoperatively. All radiological examinations were carried out in lying positions. Furthermore, the cement leakage and the corresponding location were evaluated. Secondary outcomes included the intra- and post-operative assessment of the cement properties such as end of adhesiveness, working time, curing time at the time of and after the application, and the assessment of the amount of injected cement as well as its overall tolerability [22]. In this context, other investigation criteria included the amount of injected cement, imaging and visibility of the cement, the optimal injection pressure, and the complete duration of the surgical procedure.

The overall efficacy was assessed by the clinical investigator and the patients by evidences for a kyphoplasty related infection and a revision of the relevant spine level. The safety of the IMD was evaluated regarding its overall tolerability, the registration of events, and the X-ray diagnosis. Fracture characteristics, vertebral body status, and clinical examinations have been performed.

### Statistics

Only descriptive statistical methods were used in the present trial as there was no control group. Continuous parameters are presented with their means, standard deviations (SDs), medians, first and third quartiles, and minimum and maximum values. Frequencies were calculated for categorical variables. The descriptive summary statistics are presented for continuous baseline data (e.g. age, height) at screening as well as for categorical data (e.g. gender, ethnic origin, nicotine consumption, alcohol consumption, special diet, and reason of fracture). In addition, fracture characteristics and vertebral body status were investigated.

Since no specific hypothesis was tested, no formal calculation of sample size was performed.

The descriptive summary statistics at screening, day 6 post-op, month 6 post-op, and month 12 post-op are presented for the overall VAS vertebral spine score, the ODI score, and for the 8 dimensions of the SF-36 Health Status Query. Additionally, pre- and post-differences (value at post-op visit—screening value) were investigated. The parameters assessed by a radiomorphometric evaluation and two-level X-ray images (angle of kyphosis and vertebral body index) at surgery, day 6 post-op, month 6 post-op, and month 12 post-op were summarised in the scope of descriptive statistics. Mean values of the clinical and radiological parameters during the follow-up were compared to the screening (*t* test); *p* values and 95% confidence intervals (CI) are given.

A shift analysis was performed on the cement leakage at surgery and day 6 post-op and to evaluate the overall tolerability at day 6, month 6, and month 12 post-op. The number and percentage of patients are given for the cement properties. All of the summary statistics are presented in order to assess the cement application procedure by evaluating the needle depth, needle volume, duration of intervention, and time frames of cement application. The number and percentage of patients are reported for the detectability of applied cement via X-ray at surgery, at month 6, and at month 12 post-op.

The overall efficacy was evaluated by the variables ‘any evidence for a kyphoplasty related infection’ and ‘had a revision of the relevant spinal level to be performed’ was assessed by means of shift tables for each of these two parameters at visits day 6, month 6, and month 12 post-op. For the additional secondary efficacy variable ‘BOS’, the number and percentage of patients are presented at the screening, at day 6, month 6, and month 12 post-op.

The AEs were reported by the investigators and provided in summary incidence tables for all reported AEs. The different proportions of the patients reporting the different AEs and SUEs, and treatment-related AEs were tabulated.

Summary statistics for vital signs and changes from the hospitalisation visit is displayed for all visits. The ECG results at screening and day 6 post-op (final examination) and the results of the physical examination as judged by the investigator were analysed using a shift table. Specifications of clinically relevant results by patient are given in an individual patient data listing. The number and percentage of patients who were still in hospital after the operation are reported by visit (day 4, day 5, and day 6).

Other analysis included previous and concomitant medications, anaesthetic and post-operative medications, number and percentage of patients who experienced any disease and underwent any previous surgery of the vertebral spine, and any complication in a previous vertebral spine intervention are shown.

All evaluations are provided for the SAF population.

## Results

In total, 19 of the 50 patients enrolled (38.8%) suffered from a fracture due to osteoporosis and 30 patients (61.2%) due to a trauma. The most frequently documented fracture level was ‘T12’ (14 fractures [26.4%]), followed by ‘L1’ (10 fractures [18.9%]) and ‘L2’ (9 fractures [17.0%]). The grade of the fracture was classified according to Genant et al. [23] and AO Spine classification [24]; it was mainly ‘mild’ and ‘moderate’ (24 fractures [45.3%] and 26 fractures [49.1%], respectively). Most of the fractures had a ‘wedge’ morphology (24 fractures [45.3%]). The age of the fracture ranged from 0 to 9 months with a mean of 0.7 ± 1.6 months. Regarding the vertebral body status, ‘any rear edge involvement’ was reported for most of the patients (23 patients [46.9%]), followed by ‘any vacuum phenomenon’ (11 patients [22.4%]).

The preoperative angle of kyphosis was 9.49° ± 6.17 and was statistically significantly improved postoperatively (4.14° ± 3.25; *p* ≤ 0.0001; 95% CI 3.74, 6.95) and at follow-up after 12 months (6.76° ± 4.05; *p* = 0.01; 95% CI 0.7, 5.1). Compared to preoperative (0.8° ± 0.23), the postoperative vertebra body index differed statistically significant (0.91° ± 0.08; *p* ≤ 0.005; 95% CI − 0.17, − 0.05). The difference was not statistically significant after 12 months follow-up (0.86° ± 0.1; *p* ≤ 0.1826; 95% CI − 0.14, − 0.03).

### Patient characteristics

The vast majority of patients had a medical history (47 patients [95.9%]). For three patients (6.1%), previous surgery of the vertebral spine was documented. In addition, one patient (2%) had a complication at a previous surgery of the vertebral spine. Physical examination, investigation of the vital signs, and ECG were performed and documented together with the baseline laboratory data. All 49 patients of the SAF population took non-anaesthetics prior or concomitant medication, whereas the most frequent were ‘analgesics’ (43 patients [87.8%]), followed by ‘antithrombotic agents’ (42 patients [85.7%]) and ‘agents acting on the renin-angiotensin system’ (32 patients [65.3%]). The SAF population also took anaesthetics after the surgery and postoperative medication. Frequent anaesthetics and postoperative medications included ‘muscle relaxants’ (48 patients [98%]) and ‘antibacterials for systemic use’ (35 patients [71.4%]).

### Treatment and cement properties

**Fig. 1:**
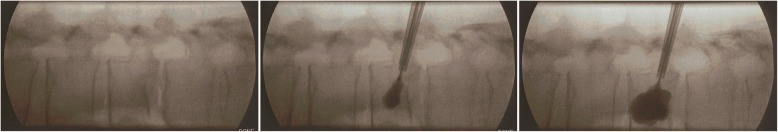
Kyphoplasty performance using image guidance X-rays. To stabilise and relatively restore the position of the fractured vertebral body ground plate, it was filled with antibiotic-loaded bone cement

Before the surgery, a mean value for angle of kyphosis of 9.5 ± 6.2° was revealed. After the surgery, smaller values were measured: 4.1 ± 3.3° at day 0, 6.2 ± 3.5° at day 6, 7.2 ± 4.8° at month 6, and 6.8 ± 4.1° at month 12. Higher mean values of the vertebral index were shown after the surgery compared to the preoperative values (0.8 ± 0.2 at day 0 pre-op, 0.9 ± 0.1 at day 0 post-op, 0.9 ± 0.1 at day 6, 0.9 ± 0.1 at month 6, and 0.9 ± 0.1 at month 12). For both parameters, the best results were revealed directly after the surgery. The results of the radiological investigation (Fig. 1) indicate an immediate and persisting stabilisation of the fractured vertebral bodies.

A mean value of 20.3 ± 1.2 °C for temperature in the operation theatre as well as the temperature of cement ready to use was revealed as this influences the success of the surgery as well [22]. The mean working duration was 4.4 ± 2.0 min. The mean balloon insufflation volume was 3.4 ± 0.9 ml on the left side and 3.3 ± 0.8 ml on the right side. For the majority of patients (28 patients [57.1%]), the overall assessment of cement properties was ‘good’ (Table 1). An ‘excellent’ overall assessment was documented for 21 patients (42.9%). The cement mixing procedure was assessed as ‘good’ for the vast majority of patients (45 patients [91.8%]).

**Table 1:** Assessment of cement properties (safety population (SAF), patients *n* = 49)

	Excellent, *n* (%)	Good, *n* (%)	Moderate, *n* (%)
Overall assessment	21 (42.9)	28 (57.1)	
Assessment of cement mixing procedure	3 (6.1)	45 (91.8)	1 (2)

**Fig. 2:**
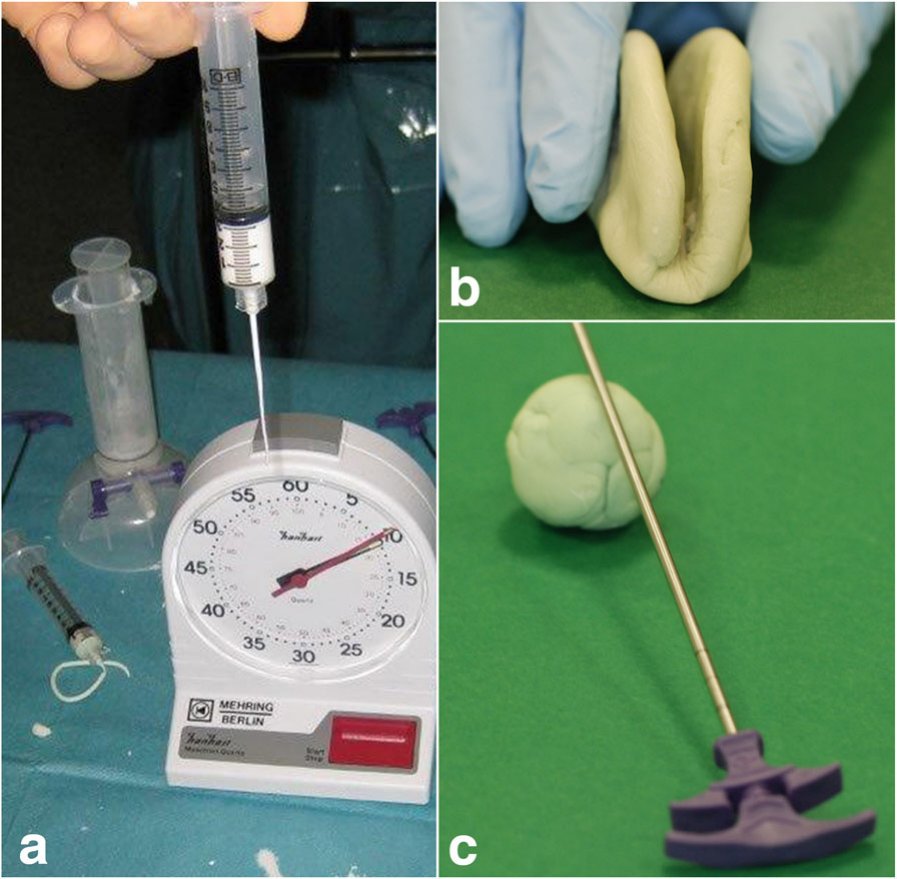
Monitoring of the cement. **a** The cement is ready to use when samples are able to form a coherent string of 10 cm (“drip” test). As soon as the cement does no longer form threads between thumb and forefinger, it can be applied immediately (“thread” test, not shown). **b** After that, and as long as folding is possible, plasticity is indicated, although the cement is not fit for usage anymore (“butterfly” test). **c** It can be assumed that the cement has hardened when contact of the sample with a metal stick provokes a certain sound (“ball” test). A previously prepared, hardened cement can be used as a reference

Figure 2 depicts the tests applied for pre- and perioperative monitoring of the cement.

Table 2 shows the evaluation of the timeframes for the cement application with a specific focus on the time of plasticity.

**Table 2:** Evaluation of the timeframes for the cement application

Application steps	Side	SAF number	Mean	Min; max
Duration of cement application	Left	49	110.74 s	30 s; 250 s
	Right	49	104.48 s	0 s; 250 s
Starting of preparation time	Left	49	29.94 s	25 s; 31 s
	Right	49	29.94 s	25 s; 31 s
Time of plasticity without adhesiveness	Left	48	6 min	4 min; 7 min
	Right	48	6 min	4 min; 7 min
Time of removal of the needle	Left	48	17 min	10 min; 25 min
	Right	48	17 min	10 min; 25 min
Time of plasticity without miscibility	Left	48	12 min	9 min; 18 min
	Right	49	12 min	9 min; 18 min
Time of loss of plasticity	Left	48	16 min	10 min; 25 min
	Right	49	16 min	10 min; 25 min
Only if leakage during surgery: time of leakage	Left	0		
	Right	1	9 min	

### Cement leakage and detectability

Cement leakage was only documented for one patient (patient no. 4). This patient had a ventral right-sided cement leakage of 0.6 ccm at surgery and at day 6. The time of leakage for the one patient with cement leakage during surgery at the right side was 9.0 min (Table 2).

The X-ray revealed an excellent detectability of the applied cement investigated for the vast majority of patients at surgery and day 6 (47 patients [95.9%] and 40 patients [81.6%], respectively). The detectability was even excellent for the majority of patients (25 patients [51.0%] and 32 patients [65.3%], respectively) at month 6 and month 12.

### Main treatment effects

One of the primary endpoints in this clinical investigation was the ‘evaluation of the function by pain scores’. Each section was scored from 0 to 5 with higher values indicating a more severe impact. For the evaluation of the disability in percent, all available items were used. The mean ODI score decreased significantly from screening with 68 ± 15.8% compared to day 6 (46 ± 19.3%; *p* < 0.0001; 95% CI 15.65, 26.74), month 6 (42 ± 21.9%; *p* < 0.0001; 95% CI 15.35, 32.72), and month 12 (43 ± 24.5%; *p* < 0.0001; 95% CI 14.3, 30.66). Congruently, the mean pre-post differences (value at post-op visit—screening value) were negative. The decreased mean ODI values after the surgery reflect a decrease of the low back pain disability and thereby an improvement on patients’ condition concerning activities of daily living. The decrease of mean ODI score was not significantly different between day 6, month 6, and month 12.

The pain level was documented using the VAS vertebral spine score. The mean VAS vertebral spine score decreased significantly from screening with 68.8 ± 17.6 to day 6 (47.6 ± 16.8; *p* < 0.0001; 95% CI 16, 25.9), month 6 (43.7 ± 20.3; *p* < 0.0001; 95% CI 13.85, 29.51), and month 12 (43.8 ± 22.2; *p* < 0.0001; 95% CI 15.3, 31.4). These results suggest an improvement of the mean pain level after the surgery compared to screening. The decrease of mean VAS vertebral spine score was not significantly different between day 6, month 6, and month 12.

**Fig. 3:**
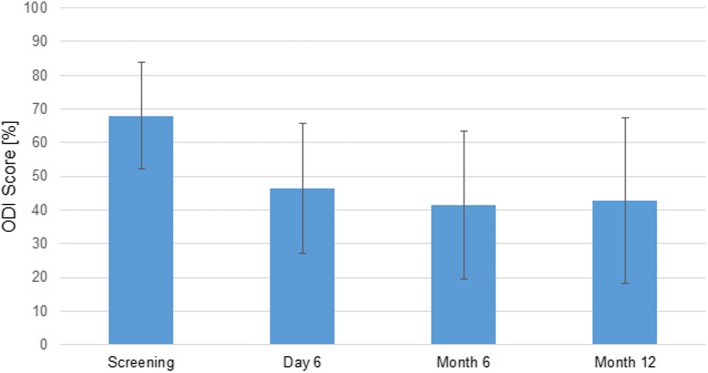
Summary statistics of the ODI score by visit (SAF, *N* = 49). Interpretation: 0–20% minimal disability, 21–40% moderate disability, 41–60% severe disability, 61–80% crippled patients, 81–100% bed-bound patients or exaggerated symptoms

**Fig. 4:**
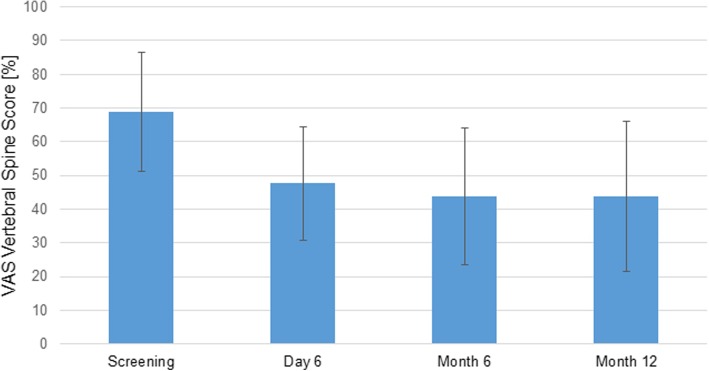
Summary statistics of the overall VAS vertebral spine score by visit (SAF, *N* = 49). A lower VAS score indicates lower pain intensity

Figure 3 shows the decrease of the low back pain disability as per ODI score. Figure 4 shows an improvement of the mean pain level after the surgery versus the screening as per VAS vertebral spine score.

In addition, an improvement of the general health state over time was shown by increasing the mean values of the SF-36 domain scores (Table 3). The highest increases of the mean value compared to screening were revealed for the domains ‘role limitations due to emotional problems’ (51.9 ± 44.7; month 6), followed by ‘role limitations due to physical health’ (36.1 ± 42.4; month 6), and ‘pain’ (34.6 ± 35.3; month 6).

**Table 3:** SF-36 domain scores

Parameter		Screening	Day 6	Month 6	Month 12
Physical functioning	Mean	16.6	34.0	39.5	37.0
	SD	20.1	21.7	26.8	28.2
	*p* value		0.0001	0.0002	0.0053
	95% CI		− 25.17; − 8.9	− 31.91; − 11.46	− 29.88;− 5.64
Role limitations due to physical health	Mean	6.6	26.0	42.6	38.2
	SD	16.8	31.4	40.3	40.9
	*p* value		0.0001	0.0002	0.0001
	95% CI		− 28.42; − 10.12	− 52.87; − 19.35	− 45.31; − 16.46
Role limitations due to emotional problems	Mean	10.2	25.7	56.8	50.0
	SD	27.4	40.8	42.2	45.9
	*p* value		0.0047	< 0.0001	< 0.0001
	95% CI		− 25.65; − 4.9	− 69.52; − 34.18	− 54.73; − 21.74
Energy/fatigue	Mean	37.2	43.0	48.9	50.3
	SD	19.4	14.7	17.7	19.2
	*p* value		0.0211	0.0227	0.0057
	95% CI		− 9.92; − 0.84	− 18.48; − 1.51	− 20.28; − 3.74
Emotional wellbeing	Mean	49.3	56.1	60.9	61.4
	SD	16.3	15.0	18.0	18.0
	*p* value		0.0098	0.0201	0.0023
	95% CI		− 10.70; − 1.55	− 14.64; − 1.36	− 14.74; − 3.5
Social functioning	Mean	45.7	54.7	68.1	65.4
	SD	28.9	26.2	22.3	26.7
	*p* value		0.0036	0.0081	0.0055
	95% CI		− 13.81; − 2.86	− 29.41; − 4.85	− 27.27; − 5.09
Pain	Mean	19.5	28.1	55.9	54.4
	SD	19.5	22.1	25.1	23.7
	*p* value		0.0132	< 0.0001	< 0.0001
	95% CI		− 14.66; − 1.8	− 48.99; − 20.93	− 44.23; − 21.80
General health	Mean	34.3	35.6	47.4	44.7
	SD	14.5	15.8	15.0	15.8
	*p* value		0.3767	< 0.0001	0.0001
	95% CI		− 4.07; 1.57	− 18.09; − 7.84	− 16.33; − 6.02

During the clinical study, four patients (8.2%) experienced at least one adverse event. The documented AEs were chronic obstructive pulmonary disease, transient ischaemic attack, bronchial carcinoma, and death. All documented serious AEs (in three patients, 6.1%) were assessed as ‘not related’ to the treatment. Two patients (4.1%) died during the course of this study: one died of bronchial carcinoma, the death of the other patient was not further specified. Both fatal adverse events were assessed as serious but had no relation to the treatment. These cases were documented as discontinuation of the patient.

## Discussion

In this monocentric, open clinical investigation in patients undergoing a one-staged kyphoplasty, the cement properties and benefits regarding application safety were evaluated, using descriptive statistical methods.

### Cement properties

The evaluation results for the primary endpoints ‘evaluation of function by pain scores’, ‘radiological investigation’, and ‘cement leakage’ revealed overall positive results. After the application of this antibiotic-loaded bone cement, the results suggest an improvement of the mean pain level after the surgery versus the screening. Furthermore, the improvement of the general health state over time as shown by increasing mean values of the SF-36 domain scores was statistically significant.

There was evidence of a decrease of the low back pain disability and thus an improvement of the patients’ condition regarding their daily activities.

This, in total, confirms other study results regarding the application of bone cement as safe and efficient in the kyphoplasty setting [25, 26].

The radiological investigation indicates an immediate and persisting stabilisation and reconstruction of the fractured vertebral bodies for the monitored study timeframe. Therefore, the applied bone cement revealed benefits in this regard. As a new aspect for bone cement suitable for vertebroplasty and/or kyphoplasty, the viscosity of the investigated material also displayed obvious benefits. This, in particular, refers to a reduced rate of leakage compared to other studies. Prior studies identified that 12.1% of the vertebral bodies had a bone cement leakage after percutaneous kyphoplasty [27] or the leakage rate was even more than 50% after vertebral body stenting and balloon kyphoplasties [28]. In this study, cement leakage was only documented for one patient (2%). This patient had a ventral right-sided cement leakage of 0.6 ccm at surgery and at day 6. Moreover, the cement used in the investigation showed proper utilisation characteristics and mostly excellent detectability (95.9% directly post-op) as well as good tolerability [29].

### Clinical effects

Concerning its overall efficacy, no infections were documented during the study for any of the patients. In addition, none of the assessed patients had a revision of the relevant spinal level between day 6 and month 6 as well as day 6 and month 12. After a 1-year follow-up period, an improved patient safety may be concluded in combination with the results outlined above. Other than the direct data evaluation of biological, chemical, and physical records throughout a study conduct the scoring systems, e.g. SF 36, ODI and others revealed some problems. Due to some patients being advanced in years, assistance was required from the clinical staff to understand and fill in the questionnaires. A second problem that can be observed in many studies was the compliance of the patients regarding the follow-up visits.

No clinically relevant abnormality was detected for vital signs at the respective visits and changes from the hospitalisation visit. Furthermore, the safety evaluation raised no safety concerns related to the kyphoplasty treatment of fractured vertebral bodies, and the antibiotic-loaded bone cement was proved to be well tolerated and safe in the evaluated kyphoplasty setting. It would be interesting to see whether the presented study findings could be verified in a larger patient group. More than that, it could be of interest to investigate those findings in a similar surgical procedure in the same field of indication like it is a vertebroplasty.

### Safety

The results of this clinical investigation showed that the kyphoplasty treatment of fractured vertebral bodies with antibiotic-loaded bone cement had a beneficial effect concerning low back pain disability, pain level, general health state of the patients, perioperative infections, and the need for a revision. Thereby, the undesirable effect of the underlying disease on patients’ daily life was in general reduced, and it was possible to achieve an improvement on patients’ condition over time. An immediate and persisting stabilisation and reconstruction of the fractured vertebral bodies was radiologically confirmed. The applied bone cement revealed proper utilisation characteristics and mainly excellent detectability as well as tolerability. The viscosity of the investigated material indicated benefits regarding a reduced rate of leakage combined with a decreased risk of infection and improved patient safety after a 1-year follow-up period. In addition, cement leakage was only documented for one patient. As an overall assessment of the cement properties for the treatment of fractured vertebral bodies, it can be stated that this bone cement could be worked homogeneously and evenly during kyphoplasty. Therefore, antibiotic-loaded bone cement excellently fulfils the requirements for bone cement in the area of kyphoplasty. The safety evaluation raised no safety concerns related to the kyphoplasty treatment of fractured vertebral bodies with the investigated medical device. It was well tolerated and safe.

### Limitations

The present study used descriptive statistical methods to analyse properties and benefits regarding application safety of an antibiotic-loaded cement in patients undergoing a one-staged kyphoplasty. Since this study is limited to the descriptive analysis of the cement, no control was necessary in this regard. However, the investigated characteristics of the cement can only be compared to results already published in the literature. Thus, in the future, a comparative study will be helpful to directly compare the present cement with other PMMA cements on the market for further validation.

## Conclusion

The cement properties of the antibiotic-loaded bone cement applied in this setting has revealed significant benefits regarding the leakage rate, alleviation of pain, and reconstruction of the structure of the vertebral body, as well as an adequate working time with a suitable viscosity for the application of bone cement in the setting of a kyphoplasty. It was possible to confirm the application safety of this investigated medical device. Finally, it may be concluded that the application technique in combination with the cement properties may guarantee the success of the surgery.

## Data Availability

The datasets used and/or analysed during the current study are available from the corresponding author on reasonable request.

## Abbreviations

ALBC: Antibiotic-loaded bone cement
ECG: Electrocardiogram
IMD: Investigational medical product
ODI: Oswestry Low-Back-Pain
OP: Operation
PMMA: Polymethylmethacrylate
SAF: Safety
SD: Standard deviation
SF-36: Short Form-36
VAS: Visual analogue scale
